# Artificial Intelligence-Generated Versus Professional Society Patient Education Materials in Gastroenterology, Surgery, Ophthalmology, and Anesthesiology: A Comparative Analysis of Readability and Health Literacy Metrics

**DOI:** 10.7759/cureus.110481

**Published:** 2026-06-08

**Authors:** Shivam Chandra, Abhin Sapkota, Vineet Kumar, Steven R Bonomo, Scott Schimpke

**Affiliations:** 1 Department of General Surgery, Rush University Medical Center, Chicago, USA; 2 Department of Internal Medicine, John H. Stroger, Jr. Hospital of Cook County, Chicago, USA; 3 College of Natural Science, Michigan State University, East Lansing, USA; 4 Department of Surgery, John H. Stroger, Jr. Hospital of Cook County, Chicago, USA; 5 Department of Surgery, Rush University Medical Center, Chicago, USA

**Keywords:** artificial intelligence, health literacy, medical specialties, patient education, readability

## Abstract

Aim: Professional society patient education materials frequently exceed recommended literacy levels, limiting equitable health information access. This study aimed to compare the readability, information quality, understandability, and actionability of artificial intelligence (AI)-generated patient education materials versus professional society materials across gastroenterology, surgery, ophthalmology, and anesthesiology.

Subjects and methods: We conducted a cross-sectional comparative analysis of 100 paired topics (25 per specialty), comparing professional society materials with the responses generated by ChatGPT (OpenAI, San Francisco, California, United States) under standardized conditions. Readability was assessed using the Flesch-Kincaid grade level, information quality with DISCERN, and understandability and actionability with the Patient Education Materials Assessment Tool (PEMAT). Paired two-sided t-tests assessed within-specialty differences.

Results: In surgery, AI-generated materials had lower reading levels and higher quality, understandability, and actionability (all p<0.001). In anesthesiology, AI materials were more readable (p<0.001) with no differences in other measures. In ophthalmology, AI improved readability (p<0.001), while professional society materials had higher quality and understandability (p<0.01) with no difference in actionability. In gastroenterology, AI materials had higher reading levels (p<0.001) with no differences in quality or usability.

Conclusion: The performance of AI-generated patient education materials varied by specialty and appeared to depend on the structure and complexity of clinical content. AI improved readability in several domains, but these gains were not uniform across specialties, particularly in areas requiring more complex or longitudinal explanations.

## Introduction

Patients increasingly rely on online resources to understand medical conditions, diagnostic testing, and treatment options. National professional societies publish patient-directed educational materials intended to provide accurate and accessible information [1,2]. However, many online materials exceed recommended readability levels, which may limit comprehension among individuals with lower health literacy [3,4]. The American Medical Association recommends that patient education materials be written at approximately a sixth- to eighth-grade reading level to improve accessibility and reduce disparities in understanding [3,5].

Large language models have emerged as an additional source of medical information for the public [6]. Conversational systems such as ChatGPT (OpenAI, San Francisco, California, United States) can generate explanations in response to patient-style questions and are now frequently used outside of clinical settings [7]. Prior studies have suggested that language model responses may be rated as comparable to physician-written answers and, in some cases, easier to read [8]. However, most evaluations have focused on single specialties or have compared artificial intelligence (AI)-generated responses with clinician-generated content rather than with patient education materials published by national professional organizations [9-11].

Educational content differs across specialties. Surgical patient education often emphasizes procedural steps, perioperative expectations, and complications, whereas gastroenterology frequently involves chronic disease management, pathophysiology, and longitudinal care [12]. These differences may influence readability and overall quality metrics. Whether AI-generated materials perform similarly across disciplines when compared with established professional society resources remains uncertain.

The objective of this study was to evaluate the readability, information quality, understandability, and actionability of patient education materials generated by a large language model compared with materials published by national gastroenterology, anesthesiology, ophthalmology, and surgical organizations (American Academy of Ophthalmology 2026; American College of Surgeons 2026; American Gastroenterological Association 2026; American Society of Anesthesiologists 2026). We sought to determine whether differences between AI-generated and organization-authored materials varied by specialty.

## Materials and methods

This cross-sectional comparative study evaluated differences in readability, information quality, understandability, and actionability between AI-generated and professional society patient education materials across four medical specialties: gastroenterology, surgery, ophthalmology, and anesthesiology. The study design was established a priori. The number of topics per specialty was predetermined at 25 to allow balanced cross-specialty comparison while maintaining feasibility. The study followed previously published methodologies used to evaluate online health information and adhered to the Strengthening the Reporting of Observational Studies in Epidemiology (STROBE) reporting guideline for cross-sectional studies [13]. This study analyzed publicly available online materials and did not involve human participants, patient data, or identifiable information. Institutional review board approval and informed consent were therefore not required. The study was conducted in accordance with the ethical principles outlined in the Declaration of Helsinki.

Patient education topics were identified from publicly available patient education libraries of nationally recognized professional societies, including the American Gastroenterological Association GI Patient Center, the American College of Surgeons patient education website, the American Society of Anesthesiologists patient education resources, and the American Academy of Ophthalmology patient education resources [14-17]. All materials were accessed between January 5, 2026, and January 12, 2026. Topics were selected to represent commonly encountered conditions, procedures, perioperative care topics, and chronic disease management scenarios. To ensure balanced representation across disciplines, 25 topics per specialty were selected a priori. For each topic, the corresponding webpage from the professional society served as the reference material. Only primary patient education content was included.

A standardized prompt template was used to generate responses from ChatGPT designed to simulate typical patient information-seeking queries corresponding to the same topics identified on the professional society websites. The model was instructed to generate patient education material for a general audience using the following prompt: "Create patient education material explaining [topic] for a general patient audience". We used the ChatGPT platform as a representative large language model-based conversational AI system. ChatGPT has rapidly gained attention in healthcare research, education, and clinical applications and has demonstrated widespread global adoption since its release. Given the growing interest in integrating AI into health systems, it was selected as an appropriate model for evaluation in this study.

All responses were generated between January 5, 2026, and January 12, 2026, using the publicly available free version of ChatGPT (GPT-4.0) accessed through the standard web interface. Each topic was entered in a new incognito browser session to minimize potential cross-conversation memory effects. Default system settings were used. No follow-up prompts, iterative refinements, or post-generation edits were performed. All AI outputs were copied verbatim for analysis.

All text was extracted and reformatted to remove hyperlinks, navigation elements, and formatting artifacts. Only the primary educational narrative content was included in analyses. Each professional society text and its corresponding AI-generated text constituted a paired comparison unit at the topic level.

Readability was assessed using the Flesch-Kincaid grade level formula, which estimates the US school grade required to understand a passage based on average sentence length and syllables per word [18]. Higher scores indicate greater linguistic complexity. Readability statistics were calculated using Microsoft Word for Mac Version 16 (Microsoft Corporation, Redmond, Washington, United States). Patient education materials written at a sixth- to eighth-grade reading level are generally recommended for accessibility [3,5].

Information quality was evaluated using the DISCERN instrument, a validated 16-item questionnaire designed to assess written information about treatment choices [19]. The first 15 items are rated on a 5-point Likert scale and evaluate clarity of aims, relevance, transparency of information sources, balance and bias, description of treatment benefits and risks, presentation of alternatives, and support for shared decision-making. The 16th item provides an overall quality rating. Total scores range from 16 to 80, with higher scores indicating higher quality. Scores above 63 are considered excellent [20]. The DISCERN instrument is freely accessible online and does not require formal permission for use.

Understandability and actionability were assessed using the Patient Education Materials Assessment Tool (PEMAT) for printable materials [21]. The understandability domain evaluates clarity, word choice, sentence structure, organization, and presentation of key points. The actionability domain evaluates whether the material clearly identifies actions the reader can take and provides concrete, manageable steps. Items are scored as agree, disagree, or not applicable. Scores are calculated as the percentage of applicable items marked agree. Scores of 70% or greater were considered indicative of adequate understandability and actionability [21]. The PEMAT is freely available through the Agency for Healthcare Research and Quality and does not require permission for research use.

Two independent reviewers evaluated all materials using the DISCERN and PEMAT instruments. Reviewers were blinded to the origin of each text during scoring. Scores from the two reviewers were averaged for analysis. Interrater reliability was assessed using percentage agreement and intraclass correlation coefficients (ICC).

Continuous variables were summarized using means and standard deviations. Because each ChatGPT-generated response was directly matched to a corresponding professional society patient education topic, paired analyses were performed at the topic level within each specialty cohort. Differences between ChatGPT-generated and professional society materials were evaluated using two-sided paired t-tests for continuous outcomes, including Flesch-Kincaid grade level, DISCERN total score, PEMAT understandability percentage, and PEMAT actionability percentage. Mean differences with corresponding p-values were calculated for each comparison. Statistical significance was defined a priori as a two-sided p-value of <0.05. Interrater reliability was interpreted using ICC, with higher ICC values indicating stronger agreement between reviewers. All statistical analyses were performed using RStudio (R Foundation for Statistical Computing, Vienna, Austria). Graphical inspection of paired differences was performed to confirm the approximate normality assumptions required for paired t-test analysis.

## Results

The gastroenterology, surgery, anesthesiology, and ophthalmology cohorts each included 25 paired patient education topics (100 total) (Table 1).

**Table 1:** Comparison of patient education metrics for ChatGPT vs. professional society materials Values represent mean scores across 25 paired topics per specialty. Comparisons between ChatGPT and professional society materials were performed using paired two-sided t-tests. Lower Flesch-Kincaid grade levels indicate easier readability; higher DISCERN and PEMAT scores indicate higher quality, understandability, and actionability. PEMAT: Patient Education Materials Assessment Tool

Metric	Gastroenterology website	Gastroenterology ChatGPT	Mean difference	Gastroenterology p-value	Surgery website	Surgery ChatGPT	Mean difference	Surgery p-value	Anesthesiology website	Anesthesiology ChatGPT	Mean difference	Anesthesiology p-value	Ophthalmology website	Ophthalmology ChatGPT	Mean difference	Ophthalmology p-value
Flesch-Kincaid grade level	8.72	10.39	1.67	0.00018	9.76	6.51	−3.25	<0.001	1.02E+01	8.00E+00	−2.21	<0.001	8.39	6.39	−2.00	<0.001
DISCERN score	64.33	64.11	−0.22	0.81	62.76	74.08	11.32	<0.001	74.6	72.32	−2.28	0.21	69.24	65.56	−3.68	<0.001
PEMAT understandability (%)	86.44	89	2.56	0.18	85.44	93.28	7.84	<0.001	90.08	88.56	−1.52	0.32	92.6	90.4	−2.20	0.009
PEMAT actionability (%)	76.39	78.89	2.5	0.28	81.8	90.12	8.32	<0.001	84.24	85	0.76	0.29	85.32	87.4	2.08	0.179

In gastroenterology, ChatGPT-generated materials demonstrated higher Flesch-Kincaid grade levels than professional society materials (10.39 vs. 8.72; mean difference: 1.67; p<0.001), with no significant differences in DISCERN, PEMAT understandability, or PEMAT actionability scores.

In surgery, ChatGPT-generated materials demonstrated lower reading levels (6.51 vs. 9.76; mean difference: −3.25; p<0.001) and higher DISCERN, PEMAT understandability, and PEMAT actionability scores (all p<0.001).

In anesthesiology, ChatGPT-generated materials also demonstrated lower reading levels (8.00 vs. 10.21; mean difference: −2.21; p<0.001), without significant differences in DISCERN or PEMAT metrics.

In ophthalmology, ChatGPT-generated materials demonstrated lower reading levels (6.39 vs. 8.39; mean difference: −2.00; p<0.001), whereas professional society materials demonstrated higher DISCERN (69.24 vs. 65.56; mean difference: −3.68; p<0.001) and PEMAT understandability scores (92.60% vs. 90.40%; mean difference: −2.20%; p=0.009). No significant difference in PEMAT actionability was observed.

## Discussion

In this multispecialty analysis, we compared patient education materials from national professional organizations in gastroenterology, surgery, anesthesiology, and ophthalmology with responses generated by ChatGPT using identical clinical topics. Patterns differed across specialties. ChatGPT-generated materials were written at significantly lower reading levels than professional society materials in surgery (6.51 vs. 9.76), anesthesiology (8.00 vs. 10.21), and ophthalmology (6.39 vs. 8.39). In contrast, no readability improvement was observed in gastroenterology, where AI-generated materials were written at a higher reading level than society-authored materials (10.39 vs. 8.72). Across specialties, both sources demonstrated consistently high information quality and usability, with DISCERN scores ranging from approximately 62 to 75 and PEMAT understandability and actionability scores exceeding 70% for both AI-generated and professional society materials.

Specialty-specific patterns were also observed. In surgical topics, ChatGPT demonstrated improvements across multiple health literacy domains, including lower reading levels and higher DISCERN, understandability, and actionability scores. In anesthesiology and ophthalmology, AI-generated materials were easier to read but demonstrated similar or slightly lower quality and usability scores compared with society-authored materials. In contrast, gastroenterology showed no measurable advantage of AI-generated content across evaluated metrics. Together, these findings suggest that the effect of AI-generated patient education varies across specialties rather than demonstrating uniform improvements across disciplines.

These differences likely reflect the structure and communication demands of specialty-specific patient education. Surgical, ophthalmology, and anesthesiology patient education is often procedural and episodic, emphasizing preparation, risks, perioperative expectations, and recovery [22,23]. This step-based structure may be well suited to large language models, which tend to organize information into concise, structured explanations that align with readability and usability frameworks. In contrast, gastroenterology frequently involves chronic disease management, pathophysiology, diagnostic testing, and long-term treatment strategies [24]. These topics may require more complex explanations that are difficult to simplify without increasing linguistic complexity. The higher reading level observed in AI-generated gastroenterology materials may therefore reflect the underlying complexity of chronic gastrointestinal conditions rather than a limitation of AI performance alone.

Prior studies evaluating large language model-generated patient education materials have reported findings similar to those observed in our study, particularly in the analyses of chronic disease topics such as chemotherapy cardiotoxicity and cancer, where many patient education resources were written above recommended health literacy thresholds [3,25,26]. Also, investigations examining oncology and other chronic disease topics have shown that chatbot responses are frequently written at reading levels exceeding recommended standards [24-26]. For example, analyses of chemotherapy education materials have reported mean reading grade levels of approximately 13-14 across several chatbots, reflecting college-level complexity, and studies examining conditions such as Wilms tumor have similarly found explanations written at high school or college reading levels. These findings are consistent with the pattern observed in the present study for gastroenterology topics, where AI-generated materials were written at higher reading levels than professional society resources.

What differs in the present study is that prior investigations evaluating procedural and surgical patient education materials have reported that many resources exceed recommended health literacy thresholds. Similarly, patient education materials in ophthalmology, anesthesiology, and surgery generated by AI systems such as ChatGPT have previously been reported at approximately 12th-, 10th-, and 10th-grade reading levels, respectively. In contrast, our analysis demonstrated that the AI-generated materials were written within the reading levels recommended by the American Medical Association. These findings suggest that, when appropriately prompted, AI-generated explanations may help produce more accessible, patient-centered educational materials.

The responses in the present analysis were generated using a more recent publicly available version of ChatGPT, and the ongoing development of health-focused initiatives such as ChatGPT Health suggests that improving medical communication and patient readability may be an increasing priority in newer systems. Together, these findings indicate that the performance of AI-generated patient education may depend on both the clinical domain and the evolving capabilities of newer model versions. Additionally, responses were generated using a standardized prompt template for patient education, which may have contributed to reading levels that were closer to recommended guidelines compared with prior studies that may not have used standardized prompts.

Beyond performance metrics, these findings have meaningful ethical implications. In the United States, the average reading level for adults is at the eighth-grade level; in fact, 80% of the American population reads at an eighth-grade or lower reading level [3]. Yet, in this study, professional society materials in surgery, anesthesiology, ophthalmology, and gastroenterology exceeded recommended reading levels, whereas AI-generated materials achieved grade levels within or near the recommended threshold in all specialties except gastroenterology. From an equity perspective, the ability of AI systems to produce materials aligned with national literacy standards may reduce barriers to comprehension and support more inclusive informed decision-making. However, this potential benefit is not uniform across disciplines, as no readability advantage was observed in gastroenterology. These findings suggest that the value of AI in patient education depends not on its mere use, but on context-specific requests.

This study has several notable strengths. First, it directly compared AI-generated responses with patient education materials from national professional societies using identical clinical topics, allowing topic-matched evaluation rather than indirect comparison. Second, the inclusion of four distinct medical specialties permitted the assessment of cross-specialty variability, strengthening the inference that AI performance is discipline-dependent rather than uniform. Third, the use of validated and widely adopted instruments, including the Flesch-Kincaid grade level, DISCERN, and PEMAT, provided multidimensional evaluation of readability, quality, understandability, and actionability. Fourth, standardized prompts and generation conditions were used for all AI responses, reducing variability and improving reproducibility. Finally, statistical analyses were conducted at the topic level with paired comparisons, enhancing methodological rigor and internal validity.

This study has several limitations. The cross-sectional design evaluated a single time point and a single version of a large language model, and results may change as models are updated or retrained. AI responses were generated using standardized prompts and may not fully represent how patients naturally interact with conversational systems or how responses evolve through follow-up questioning. The analysis focused on text-based materials and did not evaluate visual aids, multimedia content, or website design, which can substantially influence comprehension and real-world usability. Readability formulas such as the Flesch-Kincaid grade level assess sentence length and word complexity but do not directly measure true comprehension, cultural appropriateness, or health literacy across diverse patient populations. Although DISCERN and PEMAT are validated tools, scoring involves subjective interpretation and may be influenced by rater variability despite the use of standardized rubrics. The sample size was limited to 100 topics across four specialties and may not be generalizable to other medical disciplines, professional society websites, or other large language models. In addition, the study evaluated informational quality but did not assess factual accuracy, clinical safety, or the potential for harmful misinformation. Finally, patient outcomes, behavior change, and clinical decision-making were not evaluated; therefore, the study cannot determine whether differences in readability or quality translate into improved patient understanding or health outcomes.

## Conclusions

In this cross-specialty analysis of 100 patient education topics, the performance of ChatGPT-generated patient education materials varied by medical specialty. ChatGPT demonstrated improved readability in surgery, anesthesiology, and ophthalmology, but not in gastroenterology. Quality, understandability, and actionability metrics were generally high across both AI-generated and professional society materials, although specialty-specific differences were observed.

These findings suggest that large language models may serve as useful adjuncts for improving the accessibility of patient education materials, particularly in procedural specialties. However, their performance is context-dependent, and AI-generated materials should be implemented with expert review to ensure accuracy, safety, and clinical appropriateness.
